# H2228细胞和EML4-ALK阳性肺癌组织中*SOCS3*基因启动子区甲基化状态的研究

**DOI:** 10.3779/j.issn.1009-3419.2016.09.01

**Published:** 2016-09-20

**Authors:** 春来 刘, 永文 李, 云龙 董, 洪兵 张, 颖 李, 红雨 刘, 军 陈

**Affiliations:** 1 300052 天津, 天津医科大学总医院肺部肿瘤外科 Department of Lung Cancer Surgery, Tianjin Lung Cancer Institute, Tianjin Medical University General Hospital, Tianjin 300052, China; 2 300052 天津，天津医科大学总医院，天津市肺癌研究所，天津市肺癌转移与肿瘤微环境实验室 Tianjin Key Laboratory of Lung Cancer Metastasis and Tumor Microenvironment, Tianjin Lung Cancer Institute, Tianjin Medical University General Hospital, Tianjin 300052, China

**Keywords:** 肺肿瘤, EML4, ALK, SOCS3, DNA甲基化, Lung neoplasms, Echinoderm microtubule-associated-protein-like 4 (EML4), Anaplastic lymphoma kinase (ALK), Suppressor of cytokine signaling 3 (SOCS3), DNA methylation

## Abstract

**背景与目的:**

人类棘皮动物微管相关蛋白样4（echinoderm microtubule-associated-protein-like 4, *EML4*）和人类间变性淋巴瘤激酶（anaplastic lymphoma kinase, *ALK*）融合基因*EML4*-*ALK*是新发现的非小细胞肺癌的驱动基因，患者具有独特的临床病理生理特征，*ALK*基因下游信号通路的异常持续激活是其重要的下游信号通路，而导致其下游基因持续激活的原因不明。细胞因子信号转导抑制因子（suppressor of cytokine signaling, SOCS）是一类在细胞信号转导过程中起重要作用的负调控因子，主要通过抑制JAK蛋白酪氨酸激酶（janus protein tyrosine kinase, JAK）信号传导和转录激活因子（signal transducer and activator of transcription, STAT）即JAK-STAT等信号通路来调控细胞的增殖、分化和凋亡。肿瘤中常存在*SOCS*基因的甲基化异常导致的失活，从而导致JAK2-STAT等信号通路持续异常活化。本研究的目的在于探讨EML4-ALK阳性H2228细胞和肺癌组织中SOCS3启动子区甲基化状态。

**方法:**

甲基化特异性PCR检测EML4-ALK阳性H2228肺癌细胞及肺癌组织中SOCS3启动子区的甲基化状态，并通过测序验证。DNA甲基转移酶抑制剂5’-Aza-dC处理H2228细胞，并通过Real-time PCR和Western blot检测SOCS3的表达水平变化。

**结果:**

MSP及测序分析发现EML4-ALK阳性细胞株H2228中存在SOCS3启动子区甲基化；7例EML4-ALK阳性肺癌组织中的3例存在SOCS3启动子区甲基化，H2228细胞经过5’-Aza-dC去甲基化处理后SOCS3的表达明显增加。

**结论:**

EML4-ALK（+）的H2228肺癌细胞及部分肺癌组织中存在SOCS3启动子区的异常甲基化，可能是EML4-ALK阳性肺癌的重要发病机制。

## Abstract (en)

**Background and objective:**

The *EML4*-*ALK* fusion gene is a newly discovered driver gene of non-small cell lung cancer and exhibits special clinical and pathological features. The JAK-STAT signaling pathway, an important downstream signaling pathway of EML4-ALK, is aberrantly sustained and activated in EML4-ALK-positive lung cancer cells fusion gene, but the underlying reason remains unknown. The suppressor of cytokine signaling (SOCS) is a negative regulatory factor that mainly inhibits the proliferation, differentiation, and induction of apoptotic cells by inhibiting the JAK-STAT signaling pathway. The aberrant methylation of the *SOCS* gene leads to inactivation of tumors and abnormal activation of the JAK2-STAT signaling pathway. The aim of this study is to investigate the methylation status of the SOCS3 promoter in EML4-ALK-positive H2228 cells and lung cancer tissues.

**Methods:**

The methylation status of the SOCS3 promoter in EML4-ALK-positive H2228 lung cancer cells and lung cancer tissues was detected by methylation-specific PCR (MSP) analysis and verified by DNA sequencing. The expression levels of SOCS3 in H2228 cells were detected by Western blot and Real-time PCR analyses after treatment with the DNA methyltransferase inhibitor 5'-Aza-dC.

**Results:**

MSP and DNA sequencing assay results indicated the presence of SOCS3 promoter methylation in H2228 cells as well as in three cases of seven EML4-ALK-positive lung cancer tissues. The expression level of SOCS3 significantly increased in H2228 cells after 5′-Aza-dC treatment.

**Conclusion:**

The aerrant methylation of the SOCS3 promoter region in EML4-ALK (+) H2228 cells and lung cancer tissues may be significantly involved in the pathogenesis of EML4-ALK-positive lung cancer.

肺癌是我国发病率和死亡率增长最快，对人类健康和生命威胁最大的恶性肿瘤，总的5年生存率仅10%左右^[1-3]^。现在的研究^[4-10]^表明，表皮生长因子受体（epidermal growth factor receptor, *EGFR*）、KRAS（kirsten rat sarcoma viral oncogene, *K*-*ras*）、人类棘皮动物微管相关蛋白样4（echinoderm microtubule-associated-protein-like 4, *EML4*）和人类间变性淋巴瘤激酶（anaplastic lymphoma kinase, *ALK*）重排形成的融合基因*EML4*-*ALK*等基因是肺癌发生发展的驱动基因（driver gene），应用针对这些驱动基因及信号通路的药物进行分子靶向治疗，在取得明显疗效的同时又避免对正常细胞伤害，显示了诱人的治疗前景。*EML4*-*ALK*融合基因2007年由日本学者发现，定位于2号染色体的短臂上（2p21和2p23）^[5]^。在非小细胞肺癌（non-small cell lung cancer, NSCLC）人群中*EML4*-*ALK*阳性率约为3%-7%左右，但在女性、无或仅少量吸烟史的肺腺癌患者中*EML4*-*ALK*融合基因阳性率高达15%^[11]^。

动物实验提示*EML4*-*ALK*融合基因具有潜在致瘤性，是肺癌发生的驱动基因，但其致瘤机制尚未阐明。*EML4*-*ALK*融合基因阳性患者对表皮生长因子受体酪氨酸激酶抑制剂（EGFR-tyrosine kinase inhibitors, EGFR-TKIs）原发性耐药，而可能对ALK抑制剂有效^[12, 13]^。2011年8月美国食品药品管理局批准ALK抑制剂克唑替尼用于局部晚期或转移性ALK阳性NSCLC的一线治疗，对于ALK阳性的NSCLC患者，克唑替尼显示出了一定的治疗活性，并可延长患者的生存期，其无进展生存期为9.7个月，但是，克唑替尼与EGFR-TKI一样，使用一段时间后会产生获得性耐药，而揭示EML4-ALK的致瘤机理对EML4-ALK患者的治疗及克服耐药将产生重要作用^[6, 9]^。

细胞因子信号转导抑制因子（suppressor of cytokine signaling, SOCS）是一类在细胞信号转导过程中起重要作用的负调控因子，主要通过抑制JAK-STAT（janus protein tyrosine kinase, signal transducers and activators of transcription）等信号通路的持续激活来调控细胞的增殖、分化和凋亡^[14-16]^。由于JAK-STAT信号通路是多种肿瘤驱动基因重要下游信号通路，近年来的研究发现，SOCS启动子区异常甲基化在多种肿瘤发生发展中起重要作用，包括肝癌、胃癌、肺癌和血液系统肿瘤等，但是在*EML4*-*ALK*融合基因阳性肺癌中的情况却未见报道^[17-21]^。本研究的目的在于探讨人类*EML4*-*ALK*融合基因阳性肺癌组织和细胞中SOCS3启动子异常甲基化情况，为进一步阐明SOCS3在EML4-ALK阳性肺癌发生发展中的作用奠定基础。

## 1 材料与方法

### 1.1 细胞株及主要仪器

人NSCLC细胞株H2228细胞，A549细胞购于ATCC、人正常肺上皮细胞BEAS-2B细胞、人胚胎肾上皮细胞HEK293细胞由天津医科大学总医院，天津市肺癌研究所保存；7例ALK阳性患者来自于天津医科大学总医院肺部肿瘤外科的手术病例。RMPI1640和DMEM培养基、胎牛血清及Trizol试剂购自Life Technologies公司（Carlsbad, CA, USA）；实时荧光定量PCR试剂盒、Premix Ex TaqHotstart Version购自Takara公司（Dalian, China）；反转录试剂盒购自Promega公司（Madison, WI, USA）；QIAampDNA Mini Kit和EpiTect Bisulfite Kit购自Qiagen公司（Hilden, Germany）；甲基转移酶抑制剂5’-氮杂-2’-脱氧胞苷（5’-Aza-dC）购自Sigma-Aldrich公司（Kansas, Missouri, USA）。

### 1.2 细胞培养及药物处理

除HEK293细胞用含10%胎牛血清（fetal bovine serum, FBS）的DMEM（dulbecco's modified eagle medium）培养，其他细胞均培养于含10%FBS的RPMI-1640培养基。细胞培养于10 cm培养皿，37 ℃、5%CO_2_饱和湿度的培养箱中，0.25%胰酶-EDTA（ethylenediamine tetracetic acid）消化传代，所有实验均采用对数生长期细胞。5’-Aza-dC溶于二甲基亚砜（dimethyl sulphoxide, DMSO）溶液中，10 μmoL/L处理6孔板中细胞（每孔2×10^5^细胞），每个浓度3个孔，处理72 h。

### 1.3 组织及细胞DNA提取

细胞及组织按QIAamp DNA Mini Kit说明书步骤进行DNA提取，DNA提取后琼脂糖电泳检测DNA纯度，并用紫外分光光度计进行定量。

### 1.4 甲基化特异性PCR（methylation-specific PCR, MSP）

#### 1.4.1 基因组DNA亚硫酸盐修饰分别取800 ng

DNA按照EpiTect Bisulfite Kit（QINGEN）的说明书进行亚硫酸盐处理，反应在PCR仪上进行，处理条件：99 ℃、5 min，60 ℃、25 min，99 ℃、5 min，60 ℃、85 min，99℃、5 min，60 ℃、175 min，最后置20 ℃不超过24 h。纯化回收的DNA -20 ℃保存备用，用于甲基化特异PCR分析。

#### 1.4.2 MSP-PCR扩增和产物的凝胶纯化取20 ng

DNA为模板进行启动子目的片段的扩增。采用巢式PCR法，第一轮PCR引物序列为: SOCS3-F 5'-GATTYGAGGGGGTTTAGTTTTAAGGA-3'，SOCS3-R 5'-CCACTACCCCAAAAACCCTCTCCTAA-3'，反应条件为：95 ℃、5 min，95 ℃、30 s，60 ℃、30 s，72 ℃、30 s，设置降落PCR，每个循环降落0.5 ℃，14个循环；再加20个循环的95 ℃、30 s，53 ℃、30 s，72 ℃、30 s。第二轮PCR取1 μL第一轮PCR产物为模板进行扩增，甲基化引物序列为：M-F 5'-GGAGATTTTAGGTTTTCGGAATATTTC-3'，M-R 5'-CCCCCGAAACTACCTAAACGCCG-3'，非甲基化引物序列为U-F 5'-GTTGGAGATTTTAGGTTTTTGGAATATTTT-3'，U-R 5'-AAACCCCCAAAACTACCTAAACACCA-3'，反应条件：95 ℃、5 min，95 ℃、30 s，55 ℃、30 s，72 ℃、30 s，30个循环；72 ℃、5 min，4 ℃保存。扩增产物经2%琼脂糖电泳进行鉴定，并设以双蒸水为模板的阴性对照。

#### 1.4.3

亚硫酸盐测序上述PCR产物经回收纯化后，交由北京华大基因公司测序，根据甲基化位点的个数计算分析*SOCS3*基因启动子甲基化水平。

### 1.5 Real-time PCR

Trizol法常规提取5'-Aza-dC处理细胞总RNA，紫外分光光度仪定量，以Reverse Transcriptase Kit（Promega）将2 μg RNA反转成cDNA。20 ng cDNA为模板，与ABI SYBR Green Master Mix（ABI）及相应引物混合，置于实时荧光定量PCR仪Applied Biosystems 7900HT Fast Real-Time PCR System instrument and software（Applied Biosystems, USA）进行Real-time PCR反应。反应条件：95 ℃、20 s；40个PCR循环（95 ℃、10 s；60 ℃、20 s；72 ℃、10 s）。以PGK作为内参照，未处理样本作为矫正因子，数据采用2^-ΔΔCT^法进行分析，ΔCT=CT-CT（PGK），ΔΔCT=ΔCT（24 h、48 h、72 h）-ΔCT（0 h）（H2228、A549、H460、B2B）^[22]^。

### 1.6 蛋白免疫印迹（Western blot）

RIPA裂解收集细胞蛋白溶液，BCA法测浓度，与5×SDS-PAGE蛋白缓冲液充分混匀，100 ℃煮沸10 min使蛋白变性后，取30 μg蛋白上样量上样，进行SDS-聚丙烯酰胺凝胶（SDS-PAGE）电泳，100 V转膜65 min、5%脱脂牛奶的1×TBST中室温封闭1 h，加入鼠抗人SOCS3单克隆抗体，1:500稀释，4 ℃孵育过夜。加HRP标记的二抗，室温孵育1 h，显色曝光^[23]^。

### 1.7 统计学方法

应用SPSS 21.0统计软件进行分析，同一细胞系两组间比较采用两样本均数*t*检验，*P* < 0.05为差异有统计学意义。

## 2 结果

### 2.1 SOC3启动子区CpG岛的查找及MSP引物设计

应用USUC数据库预测人SOCS3 DNA启动子序列并获取启动子上游2, 000 bp，再通过MethPrimer在线分析网站和Methyl Primer Expressv 1.0软件分析这段序列的CPG岛，采用标准：碱基对 > 300，GC > 50%，观察值/预测值> 0.6。结果显示SOCS3启动子的CpG岛长度545 bp，位于871 bp-1, 415 bp。通过Methyl Primer Expressv 1.0软件设计出PCR引物。

### 2.2 MSP法检测细胞和肺癌肿瘤组织中SOCS3启动子甲基化状态

提取7例EML4-ALK阳性肺癌患者肿瘤组织和EML4-ALK阳性肿瘤细胞H2228、EML4-ALK阴性肿瘤细胞A549、HEK-293细胞、人胎盘细胞的DNA，应用重亚硫酸盐处理，未甲基化的胞嘧啶（C）将转变为尿嘧啶（U），而甲基化的胞嘧啶（C）不变。将上述经重亚硫酸盐处理后的DNA用MSP引物扩增，来检测SOCS3启动子区的甲基化情况。图 1所示MSP结果显示H2228细胞、A549细胞、HEK-293细胞、H460细胞和7例肿瘤组织中的3例SOCS3启动子区存在不同程度的甲基化，BEAS-2B细胞及人胎盘组织细胞中不存在SOCS3启动子区的甲基化情况。PCR产物经DNA测序分析，证实SOCS3启动子区存在DNA甲基化（图 2）。

**1:**
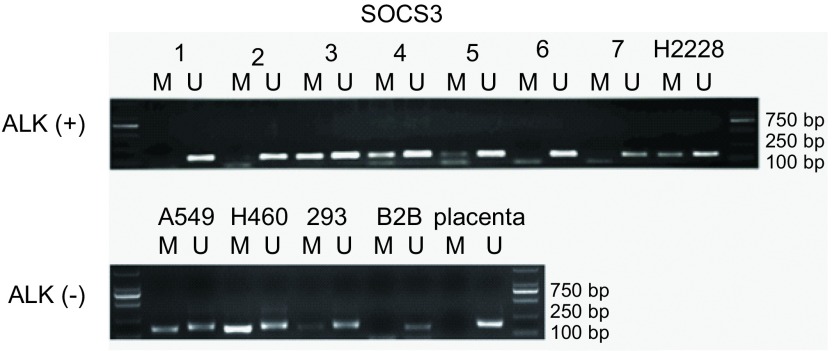
MSP方法检测肺癌细胞株及EML4-ALK阳性肺癌样品中*SOCS3*基因启动子甲基化状态。M：甲基化特异；U：非甲基化特异 MSP assay for *SOCS3* promoter in different lung cancer cell lines and EML4-ALK (+) lung cancer tissues. M:methylated-specific; U: unmethylated-specific.

**2:**
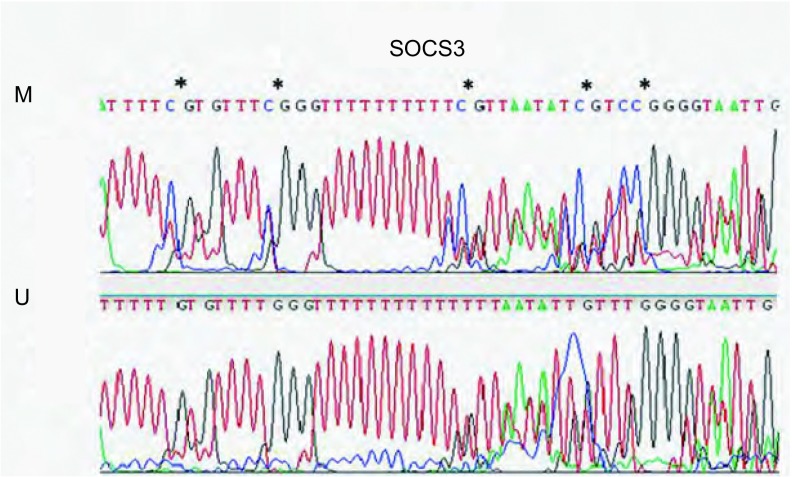
H2228细胞*SOCS3*启动子MSP分析后测序验证 The MSP result of *SOCS3* promotor were verified by DNA sequencing in H2228 cells

### 2.3

甲基转移酶抑制剂5’-Aza-dC处理，上调SOCS3表达为了进一步探讨DNA甲基化对SOCS3表达的调控情况，我们用甲基转移酶抑制剂5’-氮杂-2’-脱氧胞苷（5’-Aza-dC）处理上述细胞。以10 μm/孔连续处理不同的细胞72 h后分别提取RNA和蛋白，用Real-time PCR及Western blot检测SOCS3表达水平的变化情况，以DMSO处理组作为对照组。结果显示，与对照组相比，A549细胞和H2228细胞中SOCS3的表达水平有明显提高，分别提高了4.97倍（*P* < 0.05）和3.66（*P* < 0.05）倍。而在BEAS-2B细胞和H460细胞中SOCS3的表达则变化不明显甚至表现为降低（图 3）。进一步通过Western blot来验证去甲基化处理后SOCS3表达水平的变化，结果与Real-time PCR结果一致（图 4）。

**3:**
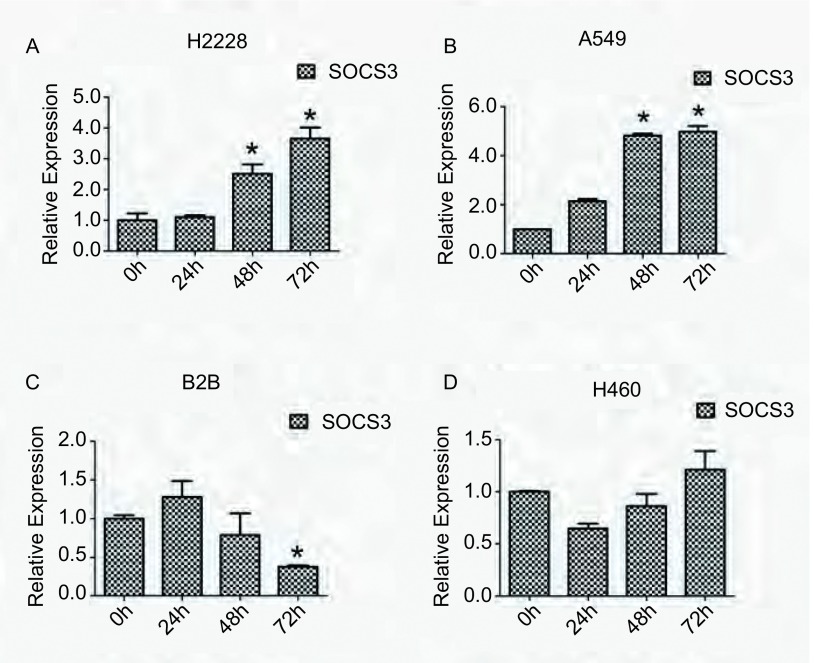
Real-time PCR检测5'-Aza-dC处理不同肺癌细胞后，SOCS3表达水平的变化情况。^*^P < 0.05 The analysis of SOCS3 expression by Real-time PCR in human lung cancer cell lines after 5'-Aza-dC treatment. ^*^P < 0.05

**4:**
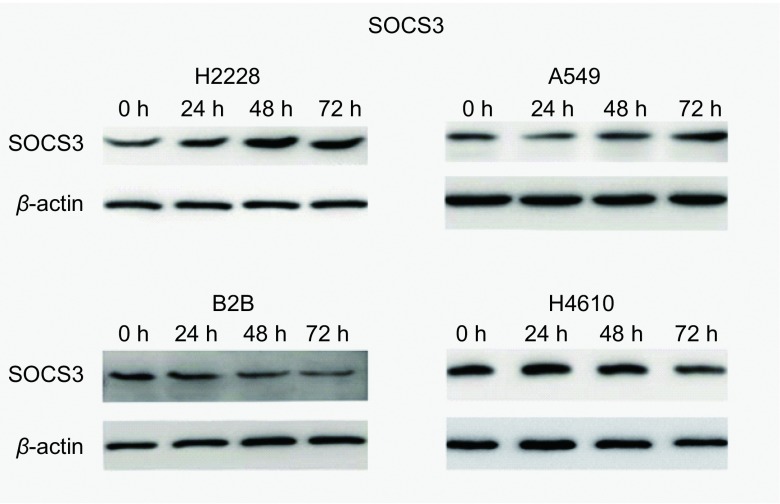
Western blot检测肺癌细胞株经5'-Aza-dC处理后SOCS3表达水平的变化 The analysis of SOCS3 expression by Western blot in human lung cancer cell lines after 5'-Aza-dC treatment

## 3 讨论

SOCS3是经典的JAK-STAT信号通路负反馈调节蛋白之一，其可以通过抑制STAT的磷酸化及其二聚体的形成或者直接抑制JAK的磷酸化来负向调节JAK-STAT通路，从而抑制细胞的持续增殖和分化^[14, 16, 24, 25]^。SOCS功能异常导致其对JAK-STAT通路的抑制作用随之消失，而启动子异常甲基化是SOCS3功能异常的重要机制。

SOCS家族分子的超甲基化在多种肿瘤中被发现，如肺癌、肝癌、前列腺癌、食管癌等^[26-29]^。比如，日本的学者用实时定量PCR和免疫印迹的方法发现肝癌组织中SOCS3的表达低于正常肝组织，并且通过特异性敲除小鼠*SOCS3*基因，使得SOCS3对STAT3的抑制作用消失导致STAT3持续活化，其抗细胞凋亡的作用随之增强，致癌物诱导肝癌发生的作用也随之增加，证明了超甲基化导致的SOCS3表达缺失与肝癌的发生有关^[20]^。

我们的研究证实在EML4-ALK阳性细胞H2228及部分EML4-ALK阳性患者肿瘤组织中存在*SOCS3*基因的异常甲基化，转甲基化酶抑制剂5’-Aza-dC处理能在mRNA水平和蛋白水平增加SOCS3的表达，MSP及基因测序分析进一步证实这些细胞中存在异常甲基化修饰。因此，在上述肺癌细胞中SOCS3的表达受异常甲基化调控。

由于SOCS蛋白在肿瘤发生发展中的作用，现在已经有学者尝试将SOCS家族蛋白激活剂用于临床前研究，并取得了一定的进展，比如，有研究^[15, 18, 30, 31]^指出肿瘤的治疗中激活SOCS后再用JAK抑制剂的效果比单用JAK激酶抑制剂的效果要好；Kim等^[30, 32, 33]^学者发现恢复SOCS1和SOCS3的表达能够增加宫颈癌细胞对放疗的敏感性；在NSCLC中也发现恢复SOCS3的表达不仅能够促进肿瘤细胞的凋亡，而且还能增加其对放疗的敏感性。因此提示针对SOCS的靶向治疗的研究，将会为肿瘤靶向治疗提供新的思路。
